# Tunisian maltreating mothers characteristics

**DOI:** 10.11604/pamj.2022.43.126.30595

**Published:** 2022-11-04

**Authors:** Soumaya Bourgou, Zeineb Azouz, Ahlem Belhadj

**Affiliations:** 1 University Tunis El Manar, Faculty of Medicine of Tunis, Rue Djebal Lakhdar 1006, Tunis, Tunisia,; 2 Child Psychiatry Department, Mongi Slim Hospital, Sidi Daoud 2046, La Marsa, Tunis, Tunisia

**Keywords:** Parenting, child abuse, maltreatment transmission, social support

## Abstract

**Introduction:**

the aim of our cross-sectional study was to compare the sociodemographic and clinical characteristics, the social support, the personal history of abuse, and the parental bonding style of maltreating Tunisian mothers to those of nonmaltreating mothers.

**Methods:**

this was a cross-sectional study carried out on the child psychiatry department of Mongi Slim Hospital in Tunisia. A data-collection sheet was designed to collect sociodemographic and clinical data about the child and the maltreatment (type, frequency, and duration), the mother of the child (sociodemographic and clinical data) and the family (socioeconomic situation and conjugal violence).

**Results:**

the sample was composed of 167 mothers. Children were significantly more maltreated when their ages were between 6 and 12 years (p=0.004) and less maltreated when they had been born prematurely (p=0.007). Also, the higher the level of the mother´s education, the less the child was maltreated (p=0.007). In addition, maltreated mothers more frequently had a history of physical abuse, emotional abuse, or emotional neglect during their childhood (p values were respectively 0.002, 0.05, and 0.007). Thus, when mothers maltreated their children, a perception of optimal grandmother-mother parenting was significantly less frequent, and a perception of an affectionless-bonding grandmother-mother was significantly frequent (p = 0.019).

**Conclusion:**

we conclude that it is important to consider individual, relational, communal, and social factors to elaborate efficient strategies for preventing children maltreatment.

## Introduction

Child abuse has always been an overlooked issue in our society and worldwide. In fact, it was in 1860 that Tardieu unveiled the first clinical descriptions of the syndrome of beaten children (Labbé, 2005). A century later, Henry Kempe readdressed the question in his book The Battered Child Syndrome [1]. As for international texts and treatises, they have appeared relatively recently. In 2002 the World Health Organization defined child abuse as: all forms of physical and/or emotional ill-treatment, sexual abuse, neglect or negligent treatment or commercial or other exploitation, resulting in actual or potential harm to the child´s health, survival, development or dignity in the context of a relationship of responsibility, trust or power [2].

Today, child abuse remains a major phenomenon all over the world. Indeed, the United Nations International Children´s Emergency Fund report, published in 2017, estimates that nearly 300 million children aged two to four years are regularly victims of violent discipline from a parent or caregiver. The report indicates also that 250 million of these children are being physically punished [3]. A variance by the perpetrator of the abuse was found in the literature [3,4]. Indeed, Ayer and co-authors reported that children with male primary caregivers were more likely to have experienced physical abuse but less likely to have experienced emotional abuse or witnessed domestic violence than children with female primary caregivers [5]. Research conducted in several countries found that mothers resorted more to physical abuse than fathers. However, fathers were the main authors of the serious wounds and even the lethal ones [6]. Most researchers examining the parental specificities in cases of maltreated children have focused on mothers and especially on maltreating mothers [7-9]. This interest may have been related to mothers being considered the primary caregiver for the child and the ones who are supposed to protect him. Maltreating the child is so “not natural”. Consequently, many authors sought to know and identify what would lead a mother to maltreat her child.

In fact, findings indicate that when compared to controls, maltreating mothers were less likely to engage in a discussion that is reflective of emotional understanding [10,11]. They also tend to have more experiential avoidance comportment with their child and maladaptive parent-child relationship processes [12]. Concerning psychiatric disorders, abusive mothers are frequently diagnosed with depression disorder, not only when they are raising their child, but also in prepartum [13,14].

Differences were also reported in hormonal secretion. In fact, Hibel *et al*. found significant differences in mother-child cortisol attunement and transmission between the maltreating and nonmaltreating groups [15]. Indeed, in his review, Strathearn reported a decreased peripheral oxytocin response to mother-infant contact in abusive-mothers cases [16]. The literature includes studies of the quality of the relationship between maltreating mothers and their parents, and its specificities, especially with respect to attachment. In this context, a meta-analysis published in 2017 included 16 studies and findings that 87.5% of abusive parents had an insecure attachment, compared to 64.5% of nonabusive parents (OR = 2.93 and p < 1 ‰) [17]. The literature has well established the connection between insecure attachment and dysfunctional parenting leading to abuse. The mediator of this link seems to be the inability to generate effective strategies for regulating emotions. Most of these results were found in European countries and America. In North African countries, studies of abuse perpetrators in general, and especially particularities, are few, as mentioned above.

We thus proposed to study the specificities of maltreating Tunisian mothers in a clinical sample by comparing them to nonmaltreating mothers. We also proposed to study their sociodemographic and clinical characteristics, their social support, their personal history of abuse, and the bonding style between them and their own parents.

## Methods

**Sample and designs:** this was a cross-sectional study carried out on a clinical population during a period of three months from April to June 2018. Our study was carried out in the child psychiatry department, the only pediatric psychiatry department located in a general hospital in Tunis, the capital of Tunisia. The Mongi Slim Hospital ethics committee agreement has been obtained under the number 1222.

**Participants:** we recruited only mothers who accompanied their children on the day of the outpatient appointment. Only consented mothers who gave their free and written consent were included. We excluded other family members who accompany the child and mothers who did not agree to participate in the study. All patients´ mothers were informed about the study. A doctor managed the participant intake of the mothers to inform them of the work, the tools, the confidential nature, and the anonymity of the collected data. They were informed that refusal to participate would not affect in any way the management of their child´s medical case, and that they could withdraw from the study at any time by refusing to complete the survey. After revision, all partially completed questionnaires were excluded.

**Assessments:** a data-collection sheet was designed to collect sociodemographic and clinical data about the child and his/her maltreatment (type, frequency, and duration), the mother of the child (sociodemographic and clinical data) and the family (socioeconomic situation and conjugal violence).

**Multidimensional Scale of Perceived Social Support (MSPSS):** we used the Arabic version of the Multidimensional Scale of Perceived Social Support [18]. The scale is composed by 12-item questionnaire to assess the perception of social support [19]. Through three subscales, the scale studied the subject´s perception of the support received from family, friends, and a significant person. A score for each subscale is calculated by dividing the total item scores of the subscale by the corresponding number of its items. The overall score is obtained by dividing the total sum of all the scores by the total number of items. The overall score thus falls between 1 and 7. According to the scores obtained, the overall social support and that of the three subscales are classified as low, medium, or high.

**Childhood Trauma Questionnaire (CTQ):** the Tunisian short version of the Childhood Trauma Questionnaire (CTQ) was used. This screening tool aims to detect experiences of childhood abuse and neglect among adults and adolescents [20]. The 28 items provide information on five subdimensions corresponding to five types of abuse: emotional abuse, physical abuse, sexual abuse, emotional neglect, and physical neglect. Each scale is presented in a 5-point Likert-type scale ranging from 5 to 25. The severity is classified into three degrees: “none to minimal,” “low to moderate,” “moderate to severe,” and “severe to extreme.” The total CTQ score takes into account the severity of the multiple forms of abuse and neglect.

**Parental Bonding Instrument (PBI):** the PBI is a 25-item self-report measure of two parenting styles: care and overprotection. It was designed for both mother and father [21]. A higher score on the “Care” subscale indicates greater parental care, and a higher score on the “Overprotection” subscale indicates greater parental control. The combined “Care” and “Overprotection” assessments allow parents to be assigned to one of the following four categories: affectionless control, affectionate constraint, optimal parenting, and neglectful parenting. Affectionless control equates to low care and high overprotection; affectionate constraint to high care and high overprotection; neglectful parenting equates to low care and low overprotection; whereas optimal parenting aligns with high care and low overprotection. In our study, we used the PBI to assess the bonding experience of each woman with both her own father and her own mother.

**Assignment criteria for the two groups:** after the data analysis, mothers were divided into two groups: group 1 (G1) and group 2 (G2) according to the existence or nonexistence of child abuse. G1 contained all maltreating mothers, whereas G2 contained non-abusive mothers. Two criteria were defined for assignment to the G1: severity and/or frequency of violence. Regarding the severity, we included and searched for all severe forms of violence. This includes acts of delivering a blow with an object or stabbing, chili in the mouth or in the anus or both, strangling, suffocation and burning, and similar forms of abuse. Psychological violence included practices deemed humiliating for the child, namely, death threats and devaluation. Regarding frequency, all types of physical and psychological abuse were considered frequent and included in the context of abuse from periodicity of at least once a month.

**Statistical analysis:** we calculated simple frequencies and relative frequencies (percentages) for qualitative variables. We calculated means and standard deviations, and we determined the extreme values for the quantitative variables. The distribution of continuous variables within the two groups was compared to a normal distribution by the Kolmogorov-Smirnov test. The comparison of two means was performed using the Student´s test for variables with a normal distribution. When the distribution of continuous variables deviated significantly from a normal distribution, the difference between groups for a continuous variable was tested using the Mann-Whitney test. Percentage comparisons were made by the Pearson Chi-square test, and if this test was invalid, Fisher´s exact bilateral test. In all statistical tests, the materiality threshold was set at 0.05.

## Results

**Groups distribution:** for three months, 179 mothers agreed to participate in our study. One hundred sixty-seven mothers completed the questionnaire. Two groups were formed according to the criteria previously described. The group of abusive mothers (G1) was formed by 108 mothers (64.7%), and the group of nonabusive mothers (G2) was formed by 59 mothers (35.3%).

Children´s profiles (Table 1): The sample of children in our study was composed of 119 boys and 48 girls with a sex ratio of 2.48.

**Table 1:** clinical and sociodemographic characteristics of maltreated children

Characteristics	G1 (N=108)	G2 (N=59)	p
**Gender**			
Male	78(72.2%)	41(69.5%)	0.709
Female	30(27.8%)	18(30.5%)	
**Age group**			
0-6 years	14(13%)	19(32.2%)	0.003***
6-12 years	71(65.7%)	25(42.4%)	0.004***
12-18 years	23(21.3%)	15(25.4%)	0.543
**Somatic problems**			
Chronic illness	30.5%	19.4%	0.106
Prematurity	1(0.9%)	4(6.8%)	0.038
**Birth order**			
First born	39 (36.1%)	17 (28.8%)	0.340
Middle child	20 (18.5%)	4 (6.8%)	0.039*
Last born	35 (32.4%)	29 (49.2%)	0.033*
Single child	14 (13%)	9 (15.2%)	0.681
**Diagnosis**			
Autism Spectrum Disorder	12 (11.1%)	18 (30.5%)	0.002**
Intellectual disabilities	23 (21.3%)	12 (20.3%)	0.884
Attention deficit hyperactivity disorder	11 (10.2%)	3 (5.1%)	0.238
Specific Learning Disorders	10 (9.2%)	6 (10.2%)	0.849
Communication Disorders	5 (4.6%)	4 (6.8%)	0.556
Motor disorders	2 (1.8%)	0 (0%)	0.293
OtherNeurodevelopmental Disorders	1 (0.9%)	0 (0%)	0.458
Depressive Disorders	7 (6.5%)	4 (6.8%)	0.941
Trauma and Stressor-Related Disorders	33 (30.5%)	12 (20.3%)	0.155
Elimination Disorders	10 (9.2%)	1 (1.7%)	0.060
Obsessive-Compulsive and Related Disorders	10 (9.2%)	1 (1.7%)	0.060
Anxiety Disorders	0 (0%)	2 (3.4%)	0.054
Disruptive, Impulse-Control and Conduct Disorders	1 (0.9%)	0 (0%)	0.458
Somatic Symptom and Related Disorders	3 (2%)	0 (0%)	0.196
Gender Dysphoria	0 (0%)	3 (5%)	0.018*
Substance-Related & Addictive Disorders	1 (0.9%)	0 (0%)	0.458
Relational Problems	1 (0.9%)	0 (0%)	0.458
No psychiatric diagnosis	3 (2.8%)	1 (1.7%)	0.662

* p < .05. **p < .01. ***p < .001.

### Mothers´ profile

#### Sociodemographic and clinical mothers´ profile (Table 2)

**Table 2:** socio-demographic characteristics of mothers

	G1	G2	p
**Age years (SD)**	40 (6)	39 (6,7)	0.344
**Age at marriage years (SD)**	26,5 (5,1)	25,8 (5)	0.457
**Educational level N (%)**			
Illiterate	6 (5.5%)	4 (6.8%)	0.752
Primary school	21 (19.5%)	6 (10.2%)	0.120
Secondary school	42 (38.9%)	16 (27.1%)	0.127
High school	32 (29.6%)	30 (50.8%)	0.007*
**Employment status N (%)**			
Employed	54 (50%)	31 (52.5%)	0.753
Unemployed	54 (50%)	28 (47.5%)	
Somatic diseases N (%)	33 (30.5%)	11 (18.6%)	0.095
**Psychiatric history N (%)**			
Bipolar disorder	2 (1.9%)	1 (1.7%)	0.942
Depressive disorders	7 (6.5%)	1 (1.7%)	0.134
Intimate partner violence N (%)	49 (45.4%)	15 (25.4%)	0.011

***Multidimensional Scale of Perceived Social Support:*** the overall score on the MSPSS questionnaire averaged 4.5 for abusive mothers and 4.6 for nonabusive mothers. No significant difference was found between the means of the overall score and the scores of the different subscales. Mothers´ perception of poor social support was more prevalent in G1: nearly 7.4% in G1 and 5.1% in G2 for the “family” subscale; 23.1% in G1 and 22% in G2 for the “friends” subscale; and 11.1% in G1 and 1.7% in G2 for the “significant person” subscale. We found a correlation between the poor quality of support by a “significant person” with the mistreatment by mothers of their children (p = 0.016).

**Figure 1:**
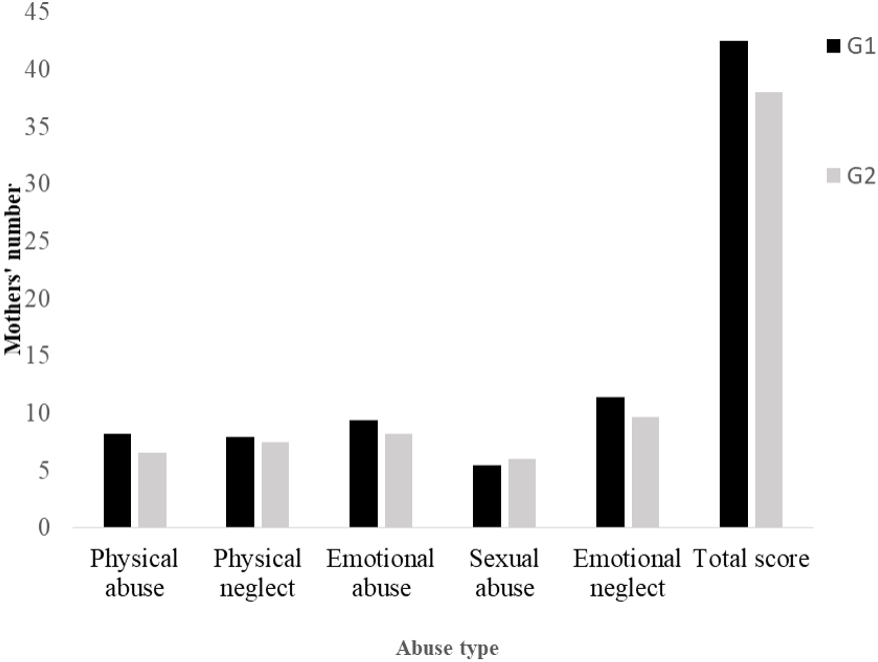
trauma during the mothers´ childhood

***Trauma during the mothers´ childhood (Figure 1):*** in total, by adding the different types of abuse and their different degrees of severity, we found that 139 mothers (83.2%) were abused during their childhood. Among these mothers, 94 in their turn abused their children. Therefore, the intergenerational transmission rate of abuse was 67.6%. Analysis of the results of the CTQ questionnaire showed that almost all the subdimensions of the CTQ questionnaire (apart from the sexual-abuse subdimension and the physical-neglect subdimension) were lower in G2. The difference was significant for the subdimensions “Physical Abuse𠇍 (p = 0.002), “Emotional Abuse” (p = 0.050), and “Emotional Neglect” (p = 0.007), as well as for the overall score (p = 0.005). The existence of severe physical abuse during childhood favoured the occurrence of maltreatment by the mother (p = 0.045). The opposite was also true since the absence of physical abuse resulted in a lower occurrence of maltreatment (p = 0.011). The lack of emotional neglect among mothers during their childhood was associated with the absence of child abuse (p = 0.031).

**Figure 2:**
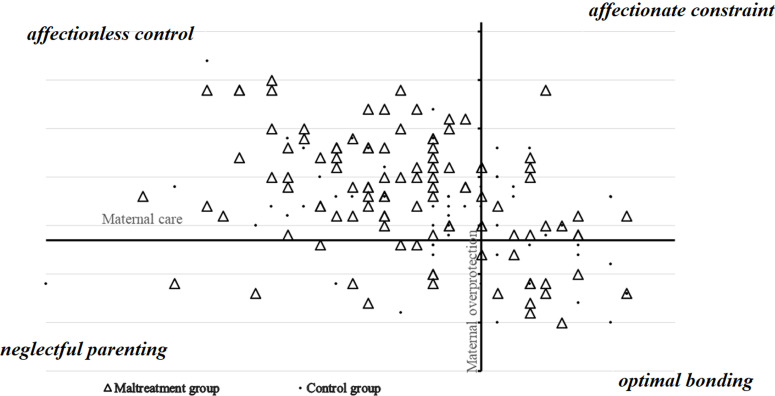
parental bonding experience of mothers

***Parental bonding experience of mothers (Figure 2):*** in our study, three mothers had never known their fathers. Thus, we were able to complete the PBI section concerning the fathers of mothers in only 164 cases. Concerning bonding to the father, no significant difference among the different types of attachment was objectified. There was no significant difference between these two dimensions for the fathers of the participating mothers. Concerning maternal bonding, the scores for the “care” and “overprotective” dimensions were respectively significantly lower (p = 0.022) and significantly higher (p = 0.039) in G1. Thus, the perception of optimal grandmother-mother parenting was significantly frequent in G2 (p = 0.037). In addition, a perception of an affectionless-bonding grandmother-mother was significantly frequent in G1 (p = 0.019).

## Discussion

There are several limitations in our work. First, the size of our clinical population was relatively small. In addition, mothers of children consulting in child psychiatry may have particular characteristics, such as psychiatric disorders that can constitute a selection bias. However, our results are similar to those in the literature. Therefore, repeating our study in the general population while increasing the sample could help us to better understand the profile of abusive mothers. Another limitation is the fact that we studied childhood abuse inflicted only by mothers. This was a methodological choice because mothers are the ones who most often accompany their children to child psychiatry consultations in Tunisia [22].

Besides all that, our work is the first Tunisian study about maltreated children. It is also the first scientific paper about the specificities of Arabic North African abusive mothers in general and about Tunisian abusive mothers especially. We found that 108 mothers in our clinical sample (64.7%) maltreated their children. We also found that maternal maltreatment depends on the demographic and clinical characteristics of both mother and child, on domestic violence, on maternal experiences of childhood maltreatment, and on bonding-style quality. Our work is also the first work using the PBI to study the bonding style between abusive mothers and their own parents.

In our study, children were victims of both physical and emotional abuse in 71.3% of cases; only physical abuse in 21.3%; only emotional abuse in 6.5%; and of a combination of neglect, physical, and emotional abuse in 0.9%. Our results are consistent with the literature, which also reported other specificities of child maltreatment, such as co-occurrences. In fact, an American study of 471 mothers found that children were exposed to neglect in 63.3% of cases, to physical abuse in 4.5%, to sexual abuse in 0.6%, and to combinations of them in 31.1% [23]. Another child-maltreatment specificity is early onset and chronicity [24]. We found that the maltreatment began on average at the age of five years and four months and lasted on average for three years and nine months.

We deduced that maternal maltreatment depended on several child variables. In fact, children were significantly more abused when they were school-aged (between 6 and 12 years) and when they attended primary schools (p = 0.004 and 0.001, respectively). Also, children under six were less frequently abused (p = 0.003). Our results contradict some studies that have shown that preschool children are the group most at risk for child abuse [25]. This difference could be explained by the fact that the Tunisian children attending the psychiatry department are mainly school-aged [22,26]. We also found that premature birth is significantly less reported in G1 (0.9%) than in G2 (6.8%) (p = 0.038). Our results are the opposite of those found in the literature. In fact, the positive link between prematurity and abuse can be explained by the fact that a premature child is generally described as being more irritable, difficult to console, and less responsive to interactions with the mother [27]. However, the traumatic experience of prematurity and the feeling of guilt could lead parents to overinvest and overprotect their children, which would protect them from abuse and thus explain our results. We found also that children from G1 had more chronic diseases than those from G2. Having a sick child could also be perceived by parents as a failure and mothers can thus develop aggressiveness, unconsciously or consciously, toward their sick child [28].

Besides the child factors cited above, we find in our study several maternal factors linked to child abuse. In fact, mothers with a university education maltreated their childless (p = 0.007). Our results are consistent with previous studies on the relationship between parents´ low educational attainment and the risk of child abuse [29]. School success influences income levels, unemployment rates, and living conditions, thus protecting against the occurrence of abuse. A history of maternal chronic somatic or psychiatric illness, and especially depressive disorder, was found more frequently in G1 than in G2. Several researchers have shown that psychiatric disorders increase two to three times the risk of the occurrence of maltreatment [30]. This risk has been explained through different hypotheses. First, mothers with a mental disorder show less sensitivity to the child´ s signals and act in a disengaged or intrusive manner. Second, the disruption of the mother-child relationship may be mediated by child behavioural disorders in response to disturbances in the maternal mood [31,32]. Third, the families of patients with mental disorders experience a high level of stress that impairs the role of a parent [31]. Another maternal factor found related to child maltreatment was exposure to domestic violence. In fact, in our study, mothers who were victims of domestic violence had abused their children significantly (p. = 0.011). Our results echo several studies that have demonstrated that domestic violence and child abuse are intimately linked and that one leads to the other [33].

In our work, we used the MSPSS scale to assess the perception of mothers of their social support. According to Cobb, social support is commonly defined as “as information leading the subject to believe that he is cared for and loved, esteemed, and a member of a network of mutual obligations”. Mothers´ perception of poor social support was more prevalent in G1 [34]. The study by Ono and Honda of 309 mother-child dyads showed that the perception of a poor level of support from family, friends, and a particular person significantly correlated with abuse (p <0.001 in the three subscales) [35]. The effect of social support on maltreatment is thought to be due to the positive effect of abuse on the well-being of parents and their mental and physical health. This involves influencing their emotions, cognitions, and behaviours [36]. Studies that have explored the link between perceptions of social support and parenting behaviours have shown that the more parents had a well-developed social network and adequate support, the better the perception of parenting skills [37]. In the literature, social support has been shown to help alleviate parental stress through a less negative assessment of stressful life events and their consequences [36]. It also promotes the consolidation of parenting skills and the psychological adjustment of mothers and provides positive feelings, and helps build self-esteem [38,39].

The difference in the two groups concerning family support did not reach the threshold of significance. This could be explained by the cultural particularity of our Tunisian population. In fact, despite changes in Tunisian society, family solidarity continues to nourish exchanges within the family. In our context, it assigns both a religious imperative and a cultural duty. Grandparents remain a great help, both in their involvement with the child and in the parental model they offer. This social particularity would help the majority of the mothers in our study, abusive or not, benefit from family support. We can conclude that even if the family is present with most mothers, it is the fact of feeling helped by a particular person, regardless of the degree of connection (family, friends, or other) that would make the difference [17,23,40]. The results of our study show that maternal antecedents of child abuse promote childbearing in adulthood. This intergenerational transmission of abuse has been widely studied in the literature. In fact, 4.99% to 54.3% of abused children become abusive parents in their turn [17,41]. Our study also confirms that intergenerational transmission is not systematic, since a history of childhood trauma was found in G2 women. However, empirical studies agree on a number of points. Some abused children will replicate the cycle of abuse in adulthood with an odds ratio ranging from 1.1 to 2.6 for mothers with a history of neglect and 1.7 to 5 for mothers with physical abuse history [11,17,23,40]. To date, various theoretical models have been advanced to explain the intergenerational transmission of maltreatment, such as the transactional ecological model of abuse [42], the attachment theory, the social learning theory and epigenetics [43,44].

In our work, we found a statistically significant correlation between child abuse and a grandmother-mother affectionless-control bonding (p = 0.019). The opposite was also true since we also found that optimal bonding was associated with the absence of child maltreatment (p= 0.037), as in the literature [45]. Thus, mothers who experienced dysfunctional relationships with their own mothers abused their children. However, no correlation was found between bonding style to father and child maltreatment. To our knowledge, no work has investigated the link between bonding to father and child mistreatment. Our result can be explained by the particularity of the relationship between Tunisian fathers and their daughters in the sixties and the seventies. In fact, the relationship between father and daughter is characterized culturally by distance and superficiality, especially in the first years of the child´s life. It appears that abuse can take root essentially from the first maternal interactions through the quality of mother-child bonding. Our work has dealt with mistreatment in clinical Tunisian populations and has shown that this is a common problem. In addition to highlighting the relatively high frequency of the problem, our study has begun to understand the factors and mechanisms that promote the occurrence of maternal abuse. In fact, it appears that maternal abuse is the result of a complex interaction between factors related to education, family and psychodynamics.

Thus, we propose the following lines of action to combat especially maternal violence against children and, in general, violence against children. First, promoting girls´ education is essential to reducing the rate of child abuse, correlated in our study with educational level. Secondly, studies have shown that there has been decrease in the prevalence of child maltreatment in countries with increased recognition and public response to the problem. This involves campaigns to raise awareness of violence. Sensitizing health care providers is also necessary, so they can so acknowledge, understand, and point to situations where a child is in danger and detect child abuse cases early. This also involves campaigns to raise awareness of violence, the introduction of sex education for children from an early age, and the early detection of proven cases of child abuse in order to start treatment early [46]. However, all these actions must be culturally appropriate. In addition, laws and dissuasive legislative measures against domestic violence must be voted on, as was recently done in Tunisia in 2017. In addition, the social support of mothers at risk for maltreatment must be improved. Many programs were implanted in many countries, including parenting classes and programs or home visits. These different programs can be difficult to implant in an Arabic country because of the conservative nature of families and their mistrust of strangers. Such programs might reduce the incidence of harsh discipline and encourage parents to improve early maternal interactions and establish a relationship of mutual respect with their children.

These different modalities, in synergy and complementarity, will facilitate in the short, medium, and long term, breaking the cycle of abuse against children. The wider significance of our study is that this wide approach to the violence problem is essential because the little girl of today will be the mother of tomorrow.

## Conclusion

Our study has made several important findings in which the intergenerational transmission of abuse is a major and determining element. The abusive mother must therefore no longer be considered merely an aggressor, but also a former victim who reproduces with her child what she has experienced herself. Our study provides important knowledge for science and practice. Our results can be used to improve existing interventions or to develop new, promising interventions. So, the prevention of violence against children is no longer just a protection for the individual but also for future generations.

### What is known about this topic

- Mothers who were abused during their childhood are at greater risk of abusing their child;
- Maltreating mothers are more likely to show signs of depression and anxiety.

### What this study adds

- Our study is the first Tunisian scientific study about maltreated children;
- We studied different specificities of maltreating mothers and the relations between them: social support, childhood maltreatment and parental bonding style.
